# Spatial Variation and Its Local Influencing Factors of Intangible Cultural Heritage Development along the Grand Canal in China

**DOI:** 10.3390/ijerph20010662

**Published:** 2022-12-30

**Authors:** Jin Yang, Lei Wang, Sheng Wei

**Affiliations:** 1 College of Architecture, Nanjing Tech University, Nanjing 211816, China; 2 Key Laboratory of Watershed Geographic Sciences, Nanjing Institute of Geography and Limnology, Chinese Academy of Sciences, Nanjing 210008, China; 3 Jiangsu Provincial Planning and Design Group Co., Ltd., Nanjing 210019, China

**Keywords:** intangible culture heritage, the Grand Canal, spatial distribution, influencing factors, geodetector

## Abstract

Understanding the spatial variation of intangible cultural heritage (ICH) is essential for protecting and utilizing heritage resources but has rarely been investigated along the Grand Canal in China. Initially, we analyzed the spatial variation of ICH with different categories using GIS spatial analysis and other technologies. Subsequently, we used the geodetector statistical method to explore local factors influencing ICH concentrations in various cities along the Grand Canal. The results show that the distribution of ICH resources in different categories was unbalanced among focal cities, mainly concentrated in the northern and southern ends of the Grand Canal. Although socioeconomic factors have important impacts on the spatial distribution of ICH, the local geographic environments remain important in forming and developing ICH resources. This study provides an important reference for ICH resource systematic regeneration and utilization plans along the Grand Canal.

## 1. Introduction

Rich intangible cultural heritage (ICH) is conducive to improving cultural soft power and enhancing national cultural self-confidence [1,2,3]. The regeneration and utilization of ICH can promote a structural transformation of the tourism industry with substantial economic benefits [4,5,6,7]. The initiation of ICH-led economic activities also forms an important factor for regional green and sustainable economic transition [8,9,10] due to ICH’s high cultural tourism value [11,12,13]. Thus, it is of great practical importance to understand the spatial distribution of ICH from a regional perspective and to provide a basis for policies of cultural heritage preservation [14].

The Grand Canal has important historical and cultural heritage values both in China and worldwide [15]. As a world heritage site [16], the Grand Canal is a hotspot in academic and practical research regarding canal-side ICH protection, inheritance, and utilization. China is currently constructing the Grand Canal National Cultural Park [17]. The protection and inheritance of ICH is an important part of the construction plan for the Grand Canal National Cultural Park [18]. Therefore, it is significant to investigate the characteristics of the Grand Canal’s cultural heritage and to assess the correlations among the project (building), site, and the Grand Canal itself [19].

Many studies have focused on the evaluation [20], protection [2], inheritance, and utilization [7] of ICH, all of which consider tourism to be a key direction for ICH development [7,21,22]. For example, ICH plays an important role in tourism development because it can increase a city’s uniqueness, deep impression, authenticity, and sense of self-discovery [23]. Therefore, ICH tourism should be regenerated and developed from the perspective of sustainable development instead of excessive commercialization [24]. In addition, some studies have focused on specific ICH categories [11,25], such as combining digital technology and drama [1]. Other studies have focused on ICH’s spatial distribution characteristics and influence mechanisms. For example, a recent study examined the coupling relationship between ICH and cultural tourism development in Hunan Province, China [14] while another study explored the spatial pattern and influencing factors of ICH on music in Xiangxi, Central China [26].

There is valuable available research about the Grand Canal and ICH development; however, few studies have combined the two from a geographical perspective. This study focuses on the spatial distribution characteristics and local influencing factors of ICH concentrations in cities along the Grand Canal. The significance of this research is threefold. First, we analyzed the spatial distribution characteristics of ICH within the core area of the Grand Canal; to our knowledge, no previous study has performed such an analysis. We used GIS spatial analysis to explore the overall spatial distribution characteristics of ICH resources and also examined the spatial distribution characteristics of different categories of ICH to understand the regional cultural differences along the Grand Canal [14]. Second, ICH formation mechanism analysis can help formulate policies according to local conditions. ICH resources are rooted in the local geographical environment in which they were cultivated, with different regional settings creating different cultural categories; therefore, the principles of classification guidance and diversity protection should always be embodied in ICH regeneration and protection [27]. Third, we provide policy suggestions for the regional coordinated development of ICH regeneration, inheritance, and utilization. As cross-regional policy research is important for China’s Grand Canal, this study attempted to present policy recommendations to promote the integrated development of ICH resources along the Grand Canal [26].

The remainder of this paper is organized as follows: Section 2 reviews the relevant literature followed by an introduction of the methodology and data sources. Section 4 presents the data analysis of the case study. Section 5 presents the discussion and conclusions.

## 2. Literature Review

There is a long history of interest in the conservation and utilization of ICH worldwide [28]. Extensive research on ICH began in 2003 with the adoption of the Convention for the Safeguarding of Intangible Cultural Heritage by United Nations Educational, Scientific, and Cultural Organization (UNESCO). Subsequent research on ICH has extended from the concept definition, identification, evaluation, classification [20], protection and inheritance [2], and utilization [7] of ICH to the impact of ICH resources on local economic and social development [29]. For example, ICH plays an important role in tourism development because it can increase a city’s uniqueness, deep impression, authenticity, and sense of self-discovery [23]. Among them, how to protect and utilize ICH effectively comprises the core issue of research in this field [30]. For example, ICH’s development of cultural tourism and creative industries is considered an effective approach [7,21,22,31]. Kim, Whitford, and Arcodia further suggest that ICH tourism should be regeneratively and sustainably developed as opposed to excessive commercialization [24].

In recent years, as the protection of ICH has received widespread attention, ICH research has gradually developed in the direction of cross-discipline and multiple methods, such as geoinformatics and statistics, which have gradually been applied to ICH research. These studies have mainly utilized GIS techniques to analyze the geographical distribution of ICH [32,33]. Methods such as kernel density analysis, nearest neighbor index, and spatial autocorrelation analysis have been used to characterize the spatial distribution of ICH at multiple scales [26,32,34,35]. The latter studies have found that the spatial distribution of ICH shows an agglomeration characteristic and tends to agglomerate in plains and hilly areas with long historic and cultural roots [14,36]. In terms of research methods, previous studies have mainly focused on qualitative descriptions, that is, by summarizing the characteristics of geographic and human environments and the cultural and ecological conditions of ICH agglomeration areas [14,27,37]. The effects of the above factors on the distribution of ICH are analyzed in the previous study. With the rapid development of information technology, methods such as ArcGIS software-based buffer analysis, overlay analysis, and spatial matching analysis have been widely used to explore the spatial relationships between ICH and various factors, such as topography, watersheds, population, roads, and economic development [33,34,36]. The results show that the spatial distribution of ICH is affected primarily by factors such as natural conditions, socioeconomic development, and historic culture and shows a clear plain orientation, river orientation, and suitable climate orientation [35].

Although existing studies have facilitated our understanding of the distribution of ICH, there are still some limitations in terms of research objects and methods of identifying driving factors. In terms of research objects, previous studies have focused on ICH along natural rivers, such as the Yellow River and the Yangtze River, while less attention has been paid to ICH along artificial canals, such as the Grand Canal in China. As a product of the interaction between humans and environments, the generation and development of ICH is closely related to the human productive and living practices that show clear regional, inherited, and social characteristics. Artificial canals facilitate economic interaction and cultural exchange among cities along the canals, which changes the daily practices in these areas and thus affects the formation and development of ICH. Therefore, the spatial distribution of ICH along artificial canals may show different characteristics from those along natural rivers. An analysis of ICH along the Grand Canal helps understand the spatial differentiation patterns of ICH along artificial canals and provides a scientific basis for the construction of the Grand Canal National Cultural Park [38,39,40,41]. In terms of methods of identifying driving factors, there has been a shift from qualitative descriptions to quantitative analysis; however, it is rare to measure the influence of different factors quantitatively, and it is difficult to compare the impacts of different factors. Recently, a limited number of studies have used the geodetector analysis method to quantitatively detect the influence intensity of different factors, such as topography, river networks, climate, economic development, transportation conditions, culture, ethnicity, and policies, on the spatial distribution of ICH [33,34], but these studies have mainly focused on the independent influences of different factors. However, the combined effect of physical geography and socioeconomic factors may differ from the influence of a single factor. Therefore, it is necessary to analyze the combined impact of different factors to identify the main driving factors of the spatial differentiation of ICH more accurately. In this regard, through the factor detection module and interaction detection module of the geodetector, this study quantitatively measures the independent and combined influence of different factors on the spatial distribution of ICH along the Grand Canal in China [35].

## 3. Materials and Methods

### 3.1. Study Area and Data Preprocessing

**Figure 1:**
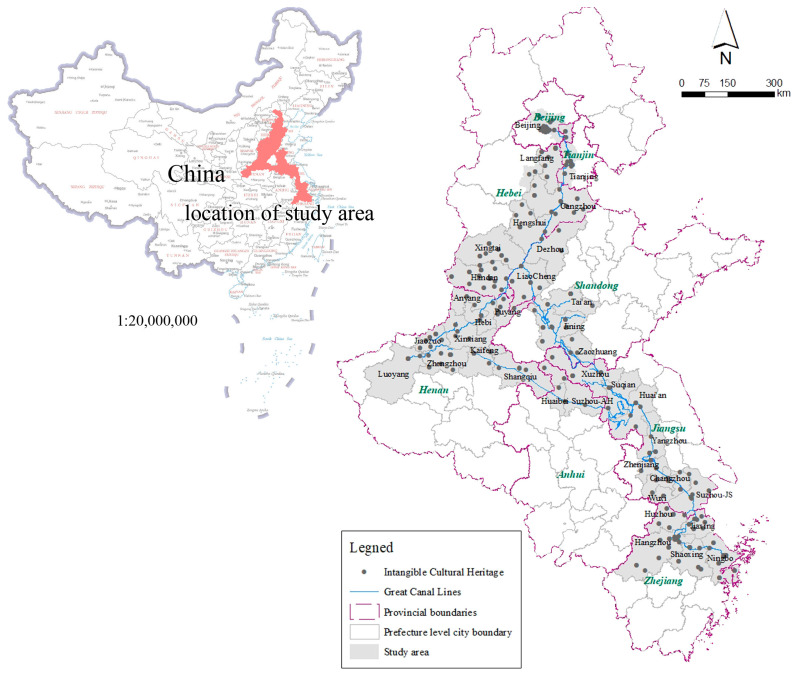
Map of the study area and the spatial distribution of ICH resources along the Grand Canal in China.

The Grand Canal connects China’s five main waterways and eight provinces with a total length of 3200 km. It has a history of more than 2500 years and was the primary route connecting northern and southern China in ancient times. The Grand Canal is situated in one of China’s wealthiest agricultural regions with a robust agricultural sector and high population density [17]. The cities along the canal contribute toward the prosperity of cities along the coastlines in China. Furthermore, the Chinese government regards 37 cities as core and expansion areas for developing the Grand Canal Cultural Belt in a series of plans (Figure 1). Thus, these 37 cities were selected as the study area for this paper, which can ensure that the research object has improved typicality and generality.

ICH formation and development results from the combined effects of the natural environment and socioeconomic development. The spatial distribution of ICH is affected by multiple factors, such as the topography, hydrology, location, population density, regional culture, and economic development level. Based on the literature and the attributes of ICH in the study area, we selected nine indicators to examine the influence factors of ICH formation and development (Table 1). We used the geographic detector method to calculate the degree of influence of different factors on the spatial distribution of ICH concentration and the effects of interactions between the two dimensions. Spatial data, such as administrative divisions, elevation, and rivers, were collected from the Resource and Environmental Science Data Center of the Chinese Academy of Sciences (http://www.resdc.cn/ accessed on 20 April 2022). In addition, the social and economic development data were collected from the statistical yearbook, the government work report, and the related official website. The ICH dataset was obtained from the China Intangible Cultural Heritage Network (http://www.ihchina.cn accessed on 20 April 2022), and the dataset provides the spatial positioning of ICH. The study area involved 617 ICH items divided into 10 categories.

### 3.2. Research Methods

#### 3.2.1. Lorentz Curve and Centralization Index

We used the Lorentz curve to characterize the degree of centralization and structural characteristics of various categories of ICH in the study area [42,43]. The Lorentz curve uses the cumulative frequency curve to assess the degree of industrialization; the degree of concentration can be inferred from the degree of convexity of the curve. The centralization index describes the degree of centralization of certain geographic data distribution, defined by the following:

(1) $$I=\frac{A-R}{M-R},$$

where *A* is the sum of cumulative percentages of different categories of ICH, *M* is the sum of cumulative percentages in the complete cluster distribution, and *R* is the sum of cumulative percentages when it is completely evenly distributed.

#### 3.2.2. Nearest Neighbor Index

On a regional scale, an ICH location can be represented as a point element in spatial geography. The spatial distribution characteristics of point elements can be quantified by the nearest neighbor index (NNI) [44,45] as follows:

(2) $$R=\frac{\bar{r}}{{\bar{r}}_{i}},$$

where $\bar{r}$ is the actual average distance between the nearest ICH sites, and ${\bar{r}}_{i}$ is the average distance of ICH sites when they are Poisson-distributed in a geographical space. The ${\bar{r}}_{i}\;$ is calculated as follows:

(3) $${\bar{r}}_{i}=\frac{1}{2\sqrt{n/A}}=\frac{1}{2\sqrt{D}},$$

where *n* is the number of ICH sites, *A* is the area of the study region, and *D* is the point density.

When *R* > 1, the point represents a feature with a uniform distribution; when *R* = 1, the point represents a feature with a random distribution; and when *R* < 1, the point represents a feature with an agglomeration distribution.

In addition, we used the z-score to estimate the aggregation significance; z-score values higher than 1.65 or lower than –1.65 were indicative of statistically significant (i.e., at the 90% confidence level) aggregations.

#### 3.2.3. Kernel Density

Kernel density estimation (KDE) [46,47] can directly reflect the spatial dispersion or agglomeration characteristics of the geographic elements. Thus, we used kernel density estimation to reflect the degree of agglomeration of ICH sites in the study area using the following:

(4) $$f\left(x\right)=\frac{1}{nh}\overset{n}{\underset{i=1}{\sum }}k\left(\frac{x-X_{i}}{h}\right),$$

where *k*() is the kernel function, h > 0 is the broadband, and $x-X_{i}$ represents the distance between the estimated value x and the ICH site $X_{i}$.

#### 3.2.4. Geodetector

Geodetector is a new statistical method for detecting spatially stratified heterogeneity and revealing the factors influencing it [48,49,50]. The principle of this method ensures its collinear immunity to multiple independent variables. The Q-statistic can be used to measure spatially stratified heterogeneity, detect explanatory factors, and analyze the interactive relationships between variables; it is calculated by Equation (5):

(5) $$q=\left(N\sigma^{2}-\overset{L}{\underset{h=1}{\sum }}N_{h}\sigma_{h}^{2}\right)/N\sigma^{2},$$

where *N* and *σ*^2^ are the sample size and variance of ICH, respectively, $N_{h}$ and $\sigma_{h}^{2}$ are the sample size and variance of category h influencing factors, respectively, and L is the classification number of influencing factors of category h. The value range of q is [0, 1] with a larger value indicating a stronger explanatory power of the index for the spatial distribution of ICH.

## 4. Results

### 4.1. The Concentration of Different ICH Categories

**Figure 2:**
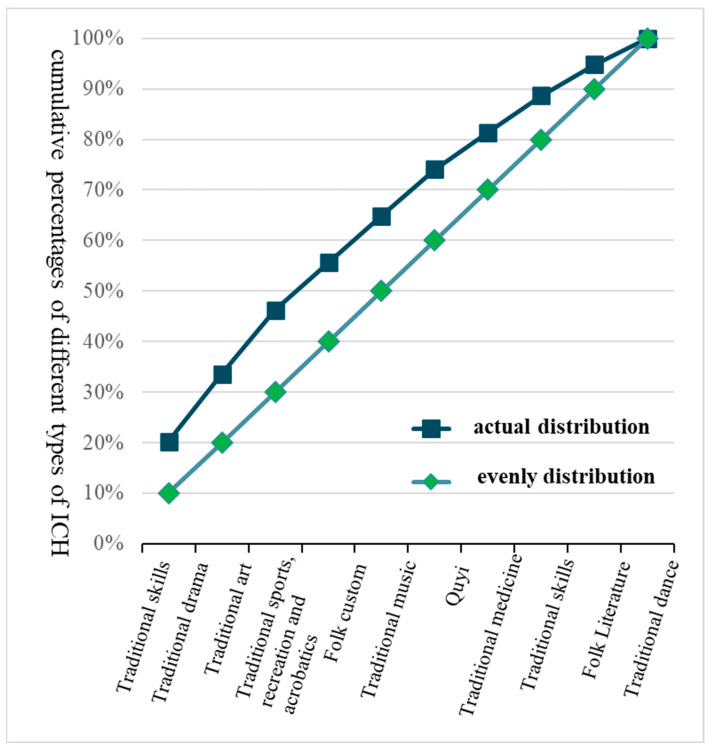
Lorenz curve distribution of intangible cultural heritage (ICH).

Figure 2 shows the Lorenz curve distribution of the 10 categories of ICH resources in comparison with a hypothetical even distribution. Among them, 125 ICH resources were in the traditional skills category, accounting for the largest proportion (20.26%) of the total number of ICH resources; 82 were in the traditional drama category, accounting for the second largest proportion (13.29%); 78 were in the traditional art category, accounting for the third largest proportion (12.64%); 58 were in the traditional sports, recreation, and acrobatics categories, accounting for the fourth largest proportion (9.40%); and 57 were in the folk customs and traditional art categories, accounting for the fifth largest proportion (9.24%). The remaining four categories of ICH, namely quyi (a type of), traditional medicine, folk literature, and traditional dance, involved 45, 45, 32, and 28 resources, and accounted for 7.29%, 7.29%, 6.16%, and 5.19%, respectively. Overall, the average number of ICH resources in each category was 10, and the average proportion of the total number of ICH resources was 10%. Only the top three categories in terms of quantity rank and proportion evaluation index exceeded the average proportion level (i.e., 10%). Combined with the smaller centralization index value of 0.243, the ICH category distribution presented an overall weak inhomogeneous structure.

### 4.2. Spatial Variation of ICH Resources among Cities

#### 4.2.1. Overall Variation of ICH Concentration among Cities

**Figure 3:**
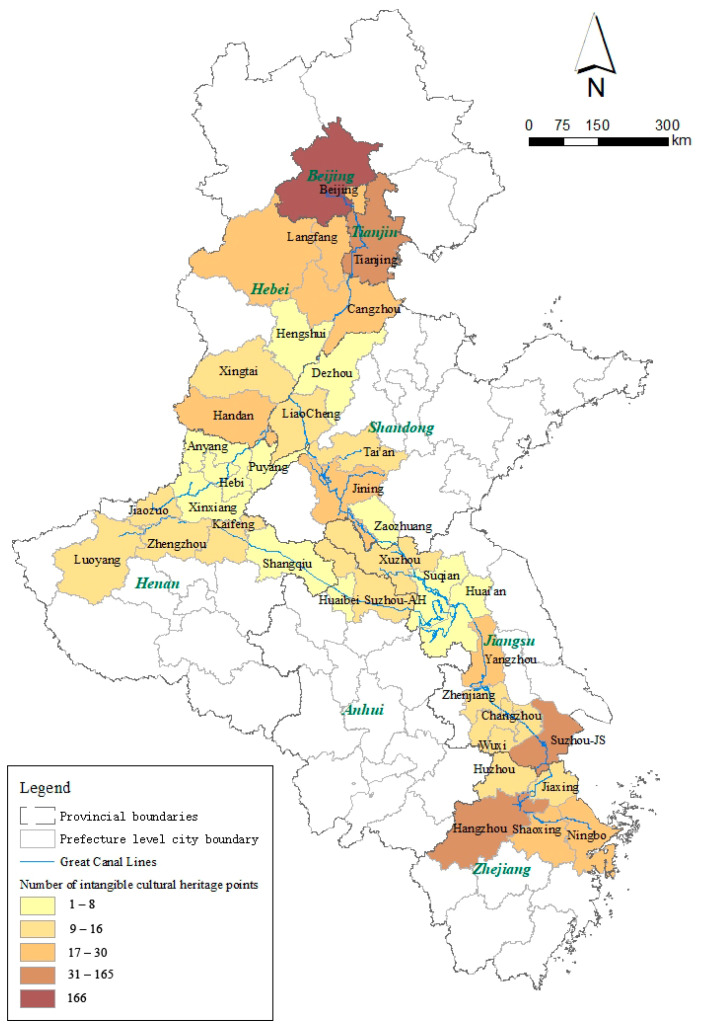
The distribution of intangible cultural heritage (ICH) resources along the Grand Canal.

**Figure 4:**
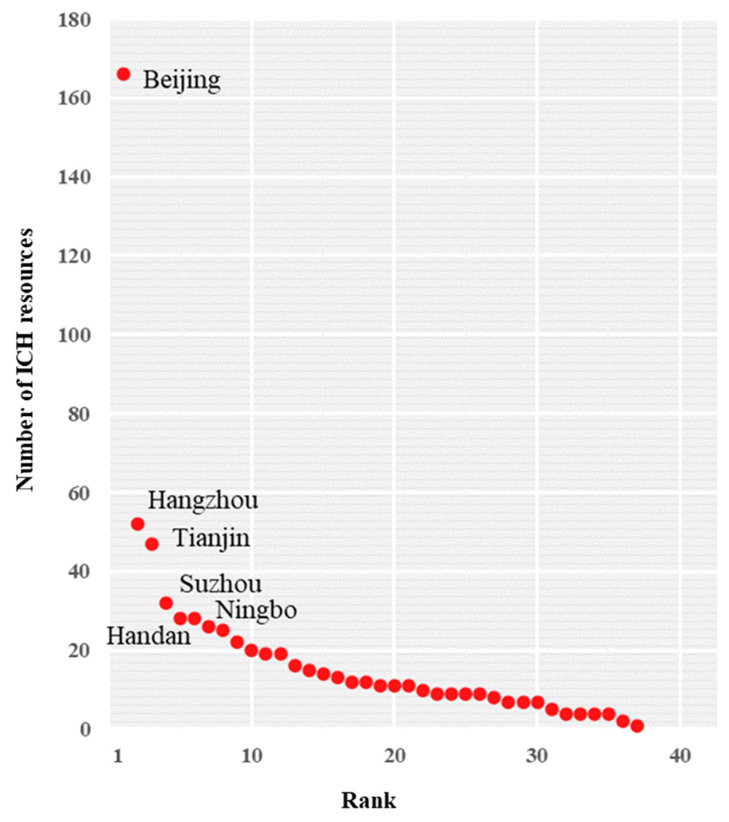
Rank sequence for the number of intangible cultural heritage (ICH) resources.

The general characteristics of spatial distribution differences were explored through two aspects: the number of ICH resources in the administrative region and the kernel density. First, based on Figure 3, the spatial distribution of ICH resources shows the characteristics of spatial differences. Beijing had the largest number of ICC resources (166), accounting for 26.9% of the total, while the ICH resources in other cities accounted for <10% of the total. There were 52 items in Hangzhou, 47 in Tianjin, and 32 in Suzhou. Overall, each city had at least one ICH resource. Beijing is China’s political, cultural, international exchange, scientific, and technological innovation center, and one of China’s historic and cultural cities and ancient capitals. Therefore, the number of ICH resources in Beijing was far greater than those in other cities. Similarly, since Hangzhou, Tianjin, Suzhou, Handan, Ningbo, and other cities also have remarkable histories of development (Figure 4), their numbers of ICH resources were greater than those of many other cities. From the perspective of geography, the numbers of ICH resources in the study area’s northern and southern administrative units were relatively large. Traditionally, the north is the pollical center while the south is the economic center of the country.

**Figure 5:**
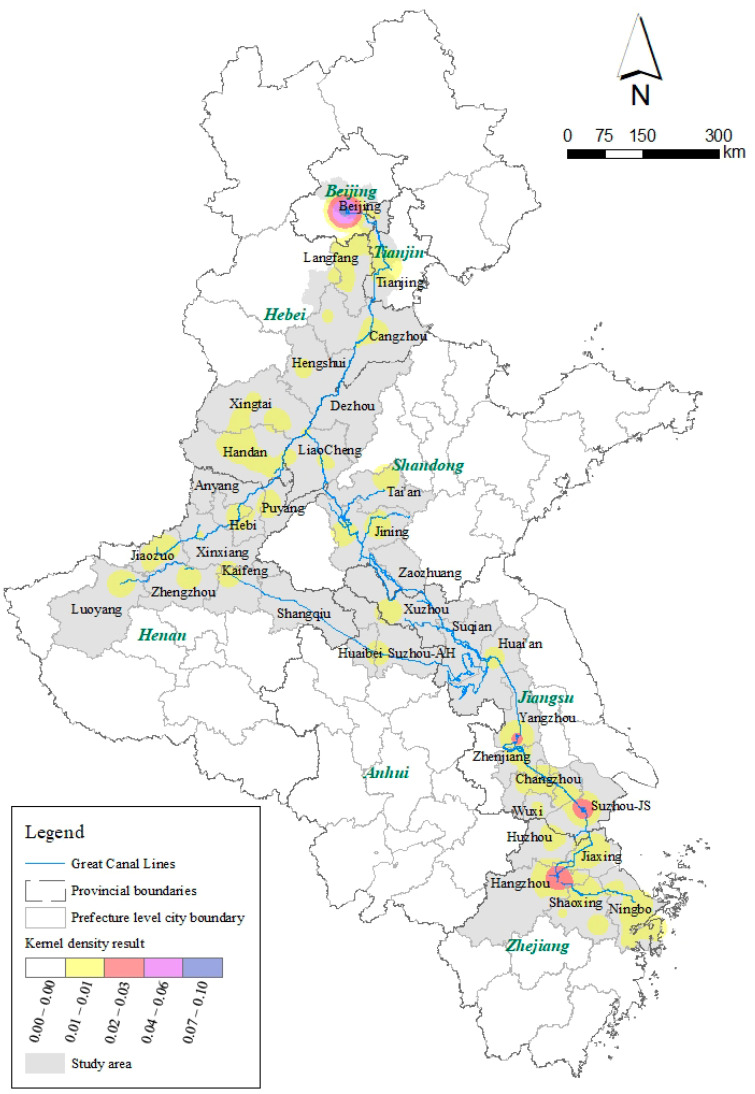
Kernel density of all intangible cultural heritage (ICH) resources.

Second, the kernel density results in Figure 5 show the overall spatial aggregation distribution characteristics of ICH resources. The aggregation characteristics of ICH in Beijing and Hangzhou are apparent, which is consistent with the overall conclusion of the quantitative distribution characteristics in those administrative regions. However, there was no obvious gathering phenomenon in Tianjin and other cities even though the numbers of ICH resources in these cities were high, as shown in Figure 3. Conversely, the ICH resources in Suzhou and Yangzhou were clustered along the Grand Canal, having greater reference values for the regeneration and utilization of the Grand Canal and ICH resources and benefitting from the geospatial proximity.

#### 4.2.2. Spatial Aggregation of Different ICH Categories among Cities

The aggregation characteristics of ICH can be obtained based on the results of the nearest-neighbor index. As shown in Table 2, the nearest-neighbor index values for all categories of ICH were 0.17 with a significance level of 99%, demonstrating an obvious clustering distribution feature. Meanwhile, several categories of ICH showed the characteristics of aggregation effects (*r*-value < 1.0); however, there were some differences among them. First, the categories with lower *R* values comprised mainly traditional skills, such as traditional medicine and art, which had higher aggregation effects with *R* values between 0.30 and 0.37. Second, traditional dance had a higher *R* value and lower aggregation state with a value of 0.70. Third, the *R* values of the other categories were between 0.40 and 0.54, which means that those categories also had strong aggregation characteristics.

**Figure 6:**
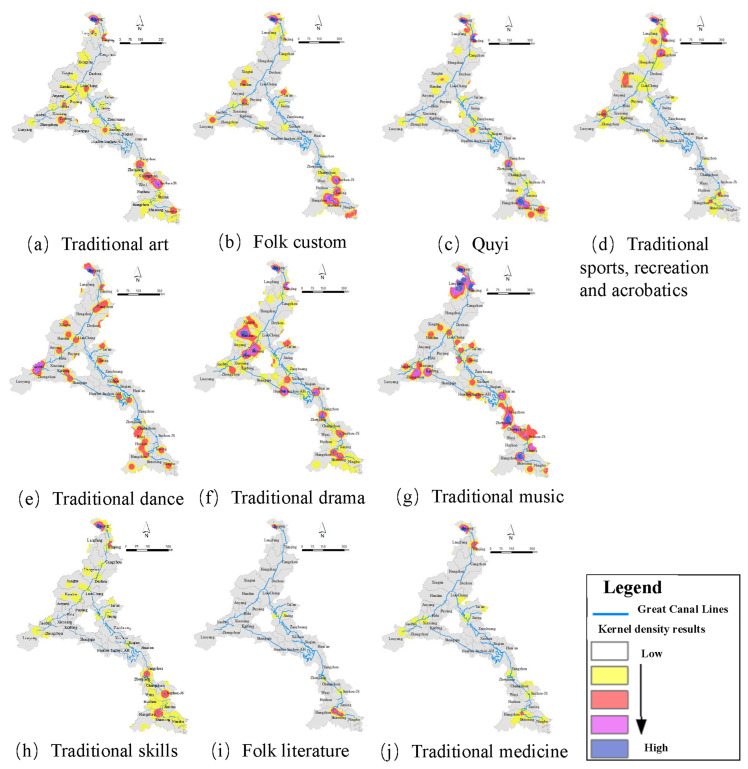
Kernel density of intangible cultural heritage (ICH) with different categories.

The kernel density results reflected more spatial distribution characteristics than those of the category perspective; three typical characteristics were observed. As shown in Figure 6a–d, the first characteristic comprised hot spots with high clustering distributed throughout the study area, mainly involving four categories of ICH, namely traditional art, folk custom, and other categories. In the long-term historical development process, these categories of ICH were closely related to the development of various regions in the study area; therefore, a certain aggregation phenomenon has formed in different regions of the study area. Second, as shown in Figure 6e–g, compared with the abovementioned four categories, there are three categories of ICH, namely traditional dance, traditional drama, and traditional music, which have formed a wider distribution of hot spots. Local inhabitants tend to develop their own distinctive content involving dance, drama, and music; therefore, the reason for this characteristic is that the area associated with the Grand Canal is broad, with many regions having unique cultural values regarding performing arts. As shown in Figure 6h–j, the third characteristic concerns hotspots in the northern and southern parts of the study area, which are more pronounced, while there is a lack of hotspots in the middle part. Three main categories of ICH involved traditional skills, folk literature, and traditional medicine; this is because these categories of ICH development are closely related to the local socioeconomic and technological development levels while these hot spots have cultivated many important economic and technical centers throughout Chinese history.

### 4.3. Local Factors Influencing Spatial Concentration of ICH among Cities

#### 4.3.1. General Pattern of ICH Concentration Determinants

The geographic detector (geodetector) analysis results showed that the influence of indicators on the spatial differentiation of ICH among canal-side cities varied (Table 3). Factors such as the number of museums (0.973), population (0.959), and economic development level (0.955) had the greatest impact on the spatial differentiation of ICH resources. Museums comprise important venues for collecting, displaying, and studying cultural heritage; their number reflects both the abundance of cultural heritage resources and the effort to protect ICH regionally. The size of the resident population demonstrates the potential for cultural exchange and the reserves of inheritors of ICH. Economic development indicates the development stage of a region. The most critical abovementioned influence factors demonstrate that ICH is generated in pursuing a higher level of cultural life after personal material needs have been satisfied. Among all factors, the influence of elevation on the spatial differentiation of ICH was the lowest with an explanatory power *q* of only 0.094, indicating that the topography of the study area had little influence on the distribution of ICH. The main reasons for this are that the Grand Canal is dominated by plains, the elevations of different cities are similar, and there is no clear topographical advantage of one city over any other. Overall, the influences of most factors of socioeconomic development were higher than those of natural geographical factors, indicating the importance of human initiatives in the development of ICH.

#### 4.3.2. Influence of Geographical Factors

Physical geographic features, such as landforms and rivers, shape local environments and influence regional cultures; along the Grand Canal, they have a certain impact on the spatial differentiation of ICH with an explanatory power *q* of 0.340 (Table 3). In our analysis, the dominant factor comprised river network density. Since ancient times, humans settled along the rivers; therefore, the distribution of ICH, which is the product of human activities, usually matches river network patterns. In the Grand Canal area, the river system plays a role in the formation and dissemination of ICH by affecting the flow of people and cultural exchange between the north and south settlements. However, the cities along the Grand Canal are all close to the coastline, and the differences regarding the influence of the water system on the distribution of ICH among different cities along the canal are small. Therefore, the explanatory power *q* of the river network density is just 0.310 (Table 3), indicating that it does not have a strong influence on the spatial distribution of ICH along the Grand Canal. Landforms with an explanatory power *q* of 0.094 had little influence on the spatial differentiation of ICH in the Grand Canal (Table 3). Although the Grand Canal spans eight provinces or municipalities, most parts comprise plains with low topography with the exception of a few areas of mountainous or hilly landforms, such as Beijing in the north, the Xingtai–Handan–Anyang–Luoyang urban region in the center, and Hangzhou and Shaoxing in the south. Flat and open space is conducive to human interaction, cultural exchange, and dissemination, thereby facilitating the formation of ICH. However, because the differences in topography among different cities in the region are small, the influence of topographical elements on the spatial differentiation of ICH among cities in the Grand Canal is not pronounced.

#### 4.3.3. Influence of Socioeconomic Factors

Socioeconomic activities create and protect ICH resources and have a critical influence on its spatial distribution. The explanatory powers *q* of the factors related to socioeconomic development on the spatial differentiation of ICH in the Grand Canal are ranked as follows (Table 3): number of museums (0.973) > resident population (0.959) > GDP (0.955) > number of traditional villages (0.720) > urbanization level (0.568) > industrialization index (0.549) > road length (0.199). First, cultural factors had the greatest influence on the spatial differentiation of ICH with the dominant factors comprising the number of museums and traditional villages. Areas with many museums and traditional villages tended to have a long history and rich culture and constitute fertile grounds for ICH. At the same time, the construction of museums and the declaration of traditional villages reflected an emphasis on cultural protection in these areas. Areas with a profound history and culture that emphasize cultural inheritance are more likely to form ICH agglomerations. Second, factors such as population and economic development significantly influence the spatial distribution of ICH. Regions with more permanent residents have more active cultural exchanges and higher human capital stock, which is conducive to protecting and developing ICH. In regions with high GDP, people have the means to pursue a higher level of cultural life and have more financial and material resources to invest in the production and inheritance of ICH. Third, the levels of urbanization and industrialization have an impact on the spatial distribution of ICH, reflecting the progress of social productivity and the modernization of industrial structures in a region. Finally, road conditions had a relatively small effect on the spatial distribution of ICH resources among cities. Convenient transportation provides important conditions for cultural exchange and dissemination, which is conducive to the formation and development of ICH; poor transportation conditions would hinder the dissemination of culture to a certain extent, but at the same time, it helps to keep ICH away from the negative impact of modern commerce in some areas, which is beneficial to the preservation of ICH. The double-side effect of transportation conditions on the development of ICH makes their impact on the spatial differentiation of ICH less obvious. In addition, in ancient times, transportation among cities along the Grand Canal was mainly based on inland water transport, which makes the spatial relationship between ICH originating in ancient times and the road network weaker, thus leading to a weak role of the road network density indicator.

#### 4.3.4. Interaction Effects of Different Factors

The detection analysis of the interaction effects for different influencing factors showed that the interaction categories are double-factor or nonlinear enhancements (Table 4). There are some differences in the interaction effects of the different factors. In general, two-dimensional factors’ influences are greater than those of one-dimensional ones. The interactions between categories of elevation and river network density with GDP, resident population, number of museums, and traditional villages are all double-factor enhancements while their separate interactions with the road length are both nonlinear enhancements. Conversely, the interaction categories of elevation and river network density with the level of urbanization and industrialization differ; the interactions of elevation with the latter two factors are both double-factor enhancements, while their interactions with river network density are both nonlinear enhancements. The interactions of river network density with most socioeconomic factors, including urbanization level, GDP, resident population, number of museums, road mileage, and industrialization level, are all strong with their explanatory powers *q* all above 0.97 (Table 4). Although the influence of a single physiographic factor, especially the topographic element, on the spatial distribution of ICH resources in the Grand Canal is weak in terms of single-factor influence, the physiographic environment, being the spatial carrier of ICH formation and development, cannot be ignored, and its impact on the formation and development of ICH needs to be considered.

## 5. Discussion and Conclusions

### 5.1. Discussion

This research has two important implications for the regeneration and utilization of Grand Canal cultural heritage. On the one hand, the scope of geographical and socioeconomic influence of the Grand Canal is very broad. An understanding of the pattern of ICH with different categories along the Grand Canal would provide evidence for the overall regeneration and utilization plan. For example, this study found that the northern and southern parts of the study area are generally more advantageous in terms of distribution characteristics of ICH whereas the central part shows a strong spatial aggregation among specific types of ICH. Therefore, protecting, inheriting, and utilizing ICH while accounting for specific types and spatial distributions are important. Some ICH resources show strong aggregations not only in place-specific areas but also in proximity to the Grand Canal. To some extent, the distance between the origin of ICH and the Grand Canal is an important factor to consider in regional development policies preserving ICH. Therefore, the spatial relationship between the Grand Canal and the kernel density of ICH is conducive to preferentially selecting appropriate ICH resources that are closer to the canal.

Second, the factors influencing ICH resource distribution were explored based on the geodetector method. In general, the influence of socioeconomic factors was more significant than that of physical geographical factors. Cultural factors had the greatest impact on the spatial differentiation of ICH with the dominant factor being the number of museums and traditional villages. Population, economic development, and other socioeconomic factors markedly influenced the spatial distributions of ICH. Factors such as urbanization and industrialization still had a notable impact while the impact of traffic was very small. Although the terrain and other single physiographical factors had weak impacts on the spatial distribution of ICH along the Grand Canal, the physical geographical environment remains an important driver in shaping the formation and development of ICH.

The results of this study further illustrate that cultural development was a core factor to cultivate ICH development. The levels of urbanization, industrialization, and transportation, among other factors, may have significant impacts on the economic and social developments of a region, but they are often not decisive for the development of ICH. The millennium development history of China’s Grand Canal indicates that it has played an important role in China’s economic and social development; cultural development on both sides of the canal is also deeply affected by it, and it infiltrates all social strata among different geographical areas. While further strengthening the regeneration and utilization of ICH, we should not only utilize its commercial benefits but also substantially protect and improve museums and facilitate the development of specific villages so that they can achieve sustainability. ICH is not only a component of Grand Canal culture but also an important carrier to highlight the cultural value of the Grand Canal. For example, the development characteristics of the Grand Canal can often be more deeply understood from the perspective of historical stories or characters [19].

The above analysis leads to policy implications on ICH regeneration and utilization. First, according to the unbalanced regional distribution of ICH, the Grand Canal’s ICH regeneration and utilization plan were formulated based on locally based factors. For example, in the regeneration and utilization of ICH in the Grand Canal, the development model “from point to area, from line to block” could be adopted, and regional cooperation should be strengthened. Second, the geographical distance between ICH resources and the Grand Canal forms an important reference for locating key exhibition areas for ICH resources. For example, to develop tourism near the Grand Canal, relevant tourism projects could be developed to showcase ICH resources close to the canal. Third, the distribution of ICH resources comprises a unified result of physiographical and socioeconomic environments; therefore, policymakers and planners should focus on the sustainable development of ICH along the Grand Canal. Fourth, a digital protection system for ICH along the Grand Canal should be implemented urgently. Specifically, we need to conduct the digital collection, storage, management, exhibition, and dissemination of the text, pictures, sound, and video materials of intangible cultural heritage and establish a dynamic resource database and a modern management service platform. Finally, constructing an identification system for the protection, inheritance, and utilization of ICH along the Grand Canal is an important foundation for further research, especially regarding the construction of interrelated cultural and tourism projects. For example, this means that tourists can easily access the icon of a relevant ICH on a map of important tourist destinations or cities within the theme of the Grand Canal and can be encouraged to understand the spatial relationships, cultural connotation, and development history behind these icons.

### 5.2. Conclusions

There were two important findings in this study. First, GIS spatial analysis and other technologies revealed that different categories characterizing ICH are nonuniformly distributed along the Grand Canal and canal-side cities. The ICH resources in some cities are relatively more concentrated. For example, ICH resources were mainly concentrated in the northern and southern ends of the canal. The pattern was closely associated with the level of development of the local economy and political status. Moreover, 10 categories of ICH resources showed different spatial distribution characteristics, which could be generally divided into three patterns. Those patterns were formed by multiscale factors, such as local environment, regional socioeconomic, and cultural attributes.

There were also some limitations to this research. First, this study only analyzed the distribution of ICH and its categories; in the future, we will be integrating more information on material cultural heritage resources [51]. Second, at the urban scale, achieving improved connections between ICH resources and the Grand Canal forms a key issue that needs to be considered from theoretical and practical perspectives. Finally, while constructing an ICH information system [52], it is necessary to consider how to integrate the location of ICH resources and the Grand Canal.

## Figures and Tables

**Table 1:** Influence factors and data sources.

Dimension	Factor	Index	Statistics		
			Max	Min	Average
Geographic factors	Landform	Elevation (m)	2262	−103	138
	River system	River network density	0.28	0.00	0.03
Social economic factors	Economic development level	GDP (billion yuan)	3610	98	614
	Population density	Permanent resident population (ten thousand people)	2189	157	696
	Location factor	Public road length (km)	24,875	4639	13,943
	Culture factor	Number of museums (number)	197	4	33
		Number of traditional villages (number)	52	0	8
	Urbanization	Urbanization level (%)	88	44	64
	Industrialization	Industrialization index	4.48	−3.50	0.00

**Table 2:** Nearest neighbor index (NNI) of intangible cultural heritage (ICH) categories.

Category	Number of Items	*r* Value	*p* Value	Z-Score
All categories	617	0.166908	0.000000	−39.588276
Traditional skills	125	0.301138	0.000000	−14.947799
Traditional art	78	0.372836	0.000000	−10.596430
Traditional sports, recreation, and acrobatics	58	0.535378	0.000000	−6.769315
Traditional dance	32	0.700168	0.001175	−3.244766
Traditional drama	82	0.535746	0.000000	−8.042542
Traditional medicine	45	0.362506	0.000000	−8.181130
Traditional music	57	0.483754	0.000000	−7.456328
Folk literature	38	0.514384	0.000000	−5.726849
Folk custom	57	0.400970	0.000000	−8.652010
Quyi	45	0.458896	0.000000	−6.944137

**Table 3:** Factors influencing spatial distribution of intangible cultural heritage (ICH) and their explanatory powers.

Dimension	Factor	Index	*q* Value		*p* Value
Geographic condition	Landform	Elevation	0.094		0.000
	River	River network density	0.310		0.000
Social economic development level	Economic development level	GDP	0.955		0.000
	Population	Permanent resident population	0.959		0.000
	Road traffic conditions	Public road length	0.199		0.000
	Culture	Number of museums	0.973	0.987	0.000
		Number of traditional villages	0.720		0.000
	Urbanization	Urbanization level	0.568		0.000
	Industrialization	Industrialization index	0.549		0.000

**Table 4:** Interactions between physical geography and social economic factors influencing ICH concentration.

		Social Economy						
		GDP	Permanent Resident Population	Public Road Length	Number of Museums	Number of Traditional Villages	Urbanization Level	Industrialization Index
Physical geography	Elevation	0.960 BE	0.963 BE	0.453 NE	0.974 BE	0.752 BE	0.633 BE	0.613 BE
	River network density	0.982 BE	0.980 BE	0.976 NE	0.979 BE	0.739 BE	0.996 NE	0.976 NE

Note: NE is nonlinear enhancement; BE is two-factor increase.

## Data Availability

The data presented in this study are available on request from the corresponding author.
